# Effects of Ospemifene on Drug Metabolism Mediated by Cytochrome P450 Enzymes in Humans *in Vitro* and *in Vivo*

**DOI:** 10.3390/ijms140714064

**Published:** 2013-07-05

**Authors:** Miia Turpeinen, Jouko Uusitalo, Terhi Lehtinen, Marita Kailajärvi, Olavi Pelkonen, Jouni Vuorinen, Pasi Tapanainen, Camilla Stjernschantz, Risto Lammintausta, Mika Scheinin

**Affiliations:** 1 Department of Pharmacology and Toxicology, University of Oulu, Oulu 90230, Finland; E-Mail: olavi.pelkonen@oulu.fi; 2 Technopolis Plc, Oulu 90590, Finland; E-Mail: jouko.uusitalo@technopolis.fi; 3 Clinical Research Services Turku, Institute of Biomedicine, Turku 20520, Finland; E-Mails: terhi.lehtinen@fimea.fi (T.L.); marita.kailajarvi@tyks.fi (M.K.); mika.scheinin@utu.fi (M.S.); 4 Pharma Ltd., Turku 20520, Finland; E-Mail: jouni.vuorinen@4pharma.com; 5 Vitabalans Ltd., Hämeenlinna 13500, Finland; E-Mail: pasi.tapanainen@vitabalans.fi; 6 Hormos Medical Ltd., Turku 20520, Finland; E-Mails: camilla.stjernschantz@fimnet.fi (C.S.); risto.lammintausta@hormos.com (R.L.)

**Keywords:** bupropion, CYP, omeprazole, selective estrogen receptor modulator, warfarin

## Abstract

The objective of these investigations was to determine the possible effects of the novel selective estrogen receptor modulator, ospemifene, on cytochrome P450 (CYP)-mediated drug metabolism. Ospemifene underwent testing for possible effects on CYP enzyme activity in human liver microsomes and in isolated human hepatocytes. Based on the results obtained *in vitro*, three Phase 1 crossover pharmacokinetic studies were conducted in healthy postmenopausal women to assess the *in vivo* effects of ospemifene on CYP-mediated drug metabolism. Ospemifene and its main metabolites 4-hydroxyospemifene and 4′-hydroxyospemifene weakly inhibited a number of CYPs (CYP2B6, CYP2C9, CYP2C19, CYP2C8, and CYP2D6) *in vitro*. However, only CYP2C9 activity was inhibited by 4-hydroxyospemifene at clinically relevant concentrations. Induction of CYPs by ospemifene in cultured human hepatocytes was 2.4-fold or less. The *in vivo* studies showed that ospemifene did not have significant effects on the areas under the plasma concentration-time curves of the tested CYP substrates warfarin (CYP2C9), bupropion (CYP2B6) and omeprazole (CYP2C19), demonstrating that pretreatment with ospemifene did not alter their metabolism. Therefore, the risk that ospemifene will affect the pharmacokinetics of drugs that are substrates for CYP enzymes is low.

## 1. Introduction

Ospemifene, a novel selective estrogen receptor modulator (SERM), was recently approved for the treatment of vulvar and vaginal atrophy (VVA) in postmenopausal women [1,2]. Phase 2 and 3 clinical trials have demonstrated the efficacy and safety of ospemifene in the treatment of VVA [1,3–6].

SERMs differ in their metabolism. Tamoxifen and toremifene are metabolized by cytochrome P450 (CYP) enzymes, whereas raloxifene is metabolized by glucuronide conjugation [7]. Ospemifene is metabolized by the liver into two major metabolites, 4- and 4′-hydroxyospemifene, which represent roughly about 70% and 7% of the total metabolites, respectively, and several minor metabolites [8]. Several cytochrome P450 enzymes, CYP2C9, CYP2C19, CYP2B6 and CYP3A4, contribute to the formation of 4-hydroxyospemifene, whereas CYP3A4 is the principal enzyme producing 4′-hydroxyospemifene. Thus, several CYP enzymes are responsible for the hepatic clearance of ospemifene. Renal clearance is a minor contributor to the overall clearance of ospemifene. Its average plasma half-life is about 20 h.

The possible effects of ospemifene on CYP-mediated metabolism were investigated as part of its preclinical and clinical development. This report presents the results of several *in vitro* and *in vivo* studies that evaluated the interaction potential of ospemifene with regard to CYP-mediated drug metabolism.

## 2. Results and Discussion

### 2.1. CYP Inhibition *in Vitro*

*In vitro*, ospemifene was a relatively potent inhibitor of CYP2B6 (IC_50_, 7.8 μM) and CYP2C9 (IC_50_, 10 μM) a less potent inhibitor of CYP2C19, CYP2C8, CYP2D6 and CYP3A4 (Table 1). Further studies indicated (results not shown) that ospemifene inhibition was competitive for CYP2B6 and CYP2D6 and likely competitive for CYP2C8. Ospemifene inhibition of CYP2C9 was competitive up to 10 μM, but a noncompetitive component emerged at higher concentrations. CYP2C19 inhibition was noncompetitive. At steady state, the mean *C*_max_ for ospemifene was found to be 785 ng/mL, *C*_min_ 94 ng/mL and *C*_av_ 227 ng/mL corresponding roughly 2 μM, 0.25 μM and 0.6 μM, respectively [9].

4-Hydroxyospemifene most potently inhibited tolbutamide methylhydroxylase activity, *i.e.*, CYP2C9 activity (IC_50_, 1.1 μM; Table 1). Inhibition of CYP2C19 activity was approximately an order of magnitude less potent. IC_50_ values for all other CYP-mediated enzyme activities were >25 μM, well above the clinical *C*_max_ of 4-hydroxyospemifene (approximately 0.3 μM for repeated oral 60-mg dosing).

4′-Hydroxyospemifene inhibited amodiaquine desethylation, *i.e.*, CYP2C8 activity, with the highest potency (IC_50_, 7 μM; Table 1). CYP2C9 and CYP2B6 were also inhibited by 4′-hydroxyospemifene, although with lesser potency. IC_50_ values for all other CYP-mediated reactions were >50 μM. The clinical *C*_max_ of 4′-hydroxyospemifene is approximately 0.1 μM for repeated oral 60-mg dosing.

Taken together, in human liver microsomal preparations *in vitro*, ospemifene showed weak inhibition potential for CYP2C9, CYP2C19, CYP2C8, and CYP2D6. Ospemifene inhibited CYP2B6 metabolism with somewhat higher potency than the other CYP enzymes, but IC_50_ values were above its *C*_max_ at therapeutic doses, indicating that ospemifene is unlikely to inhibit CYP-mediated drug metabolism. The potential of 4-hydroxyospemifene and 4′-hydroxyospemifene to inhibit CYP-mediated drug metabolism also appeared to be low. 4′-Hydroxyospemifene showed some potential for CYP2C8 inhibition, but after clinical doses of ospemifene, the *C*_max_ of 4′-hydroxyospemifene at steady state is still much lower than its IC_50_ values. Conversely, 4-hydroxyospemifene demonstrated relatively high potency to inhibit CYP2C9 activity.

### 2.2. *In Vitro* CYP Induction in Human Hepatocytes

In isolated human hepatocytes, incubation with ospemifene (0.2, 2, and 20 μM) for 48 h did not cause any relevant changes in CYP1A2, CYP2C9, or CYP2C19 activity. The highest concentration (20 μM) of ospemifene was associated with approximately two-fold induction of CYP2B6 and CYP3A4 activities. Three reference CYP inducers (omeprazole, phenobarbital, or rifampicin) induced the expected approximately 1.3- to 41.6-fold increases in the respective CYP enzyme activities (data not shown).

### 2.3. Clinical Pharmacokinetics Studies

To assess the *in vivo* effects of ospemifene on CYP-mediated metabolism, three Phase 1 crossover pharmacokinetic studies were conducted using recommended probe drugs for CYP2C9 (warfarin), CYP2B6 (bupropion) and CYP2C19 (omeprazole) [10,11].

#### 2.3.1. Study 1: The Effect of Ospemifene on Warfarin Pharmacokinetics

**Figure 1:**
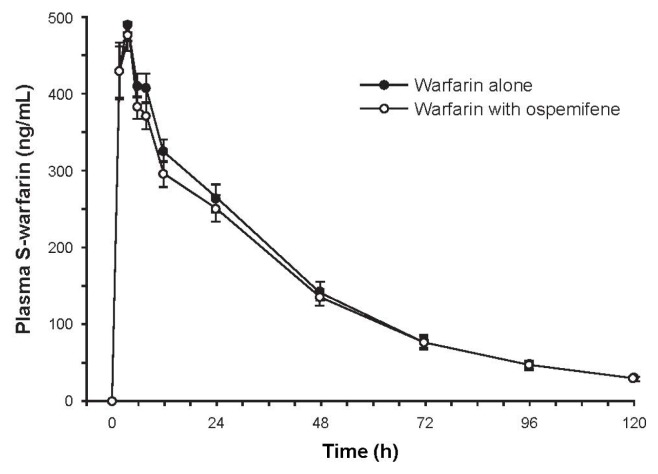
Mean concentration-time curve of plasma *S*-warfarin after administration of 10 mg of warfarin with and without ospemifene pretreatment. Error bars are ±SE, *N* = 16.

Subjects (*N* = 16) were Finnish white females with a mean age of 61.7 years (range, 52–71 years). Mean plasma concentrations of *S*-warfarin over time were similar with and without ospemifene pretreatment (Figure 1). The ratio of geometric least square means of AUC_∞_ (90% CI) between pretreatment with ospemifene *versus* without was 0.963 (0.909–1.020), indicating that ospemifene did not interfere with *S*-warfarin metabolism. Secondary pharmacokinetic parameters were similar between the two treatments (Table 2).

#### 2.3.2. Study 2: The Effect of Ospemifene on Bupropion Pharmacokinetics

**Figure 2:**
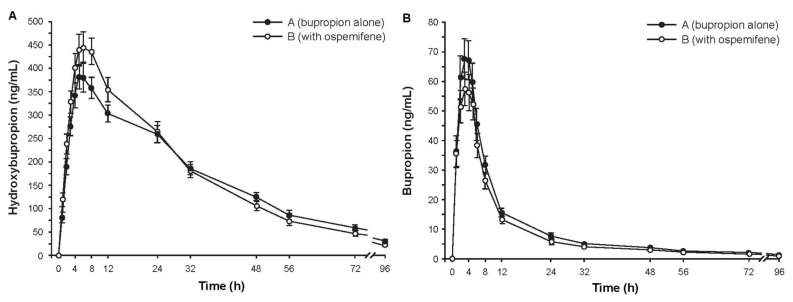
Mean concentration-time curves of plasma (**a**) hydroxybupropion and (**b**) bupropion after administration of 150 mg of bupropion with or without ospemifene pretreatment. Error bars are ±SE, *N* = 16.

Subjects (*N* = 16) were Finnish white females with a mean age of 61.9 years (range, 50–70 years). Mean plasma concentrations of hydroxybupropion over time were similar with and without ospemifene pretreatment (Figure 2A). The ratio of geometric least square means (90% CI) for hydroxybupropion AUC_∞_ with and without ospemifene pretreatment was 0.977 (0.921–1.036), indicating that ospemifene did not interfere with hydroxybupropion metabolism. Concentrations of bupropion in plasma (Figure 2B) were much smaller than those of hydroxybupropion. The mean AUC_∞_ of plasma bupropion was also similar with and without ospemifene pretreatment. Secondary pharmacokinetic parameters of hydroxybupropion and bupropion further indicated absence of a pharmacokinetic interaction (Table 2).

#### 2.3.3. Study 3: The Effect of Ospemifene on Omeprazole Pharmacokinetics

**Figure 3:**
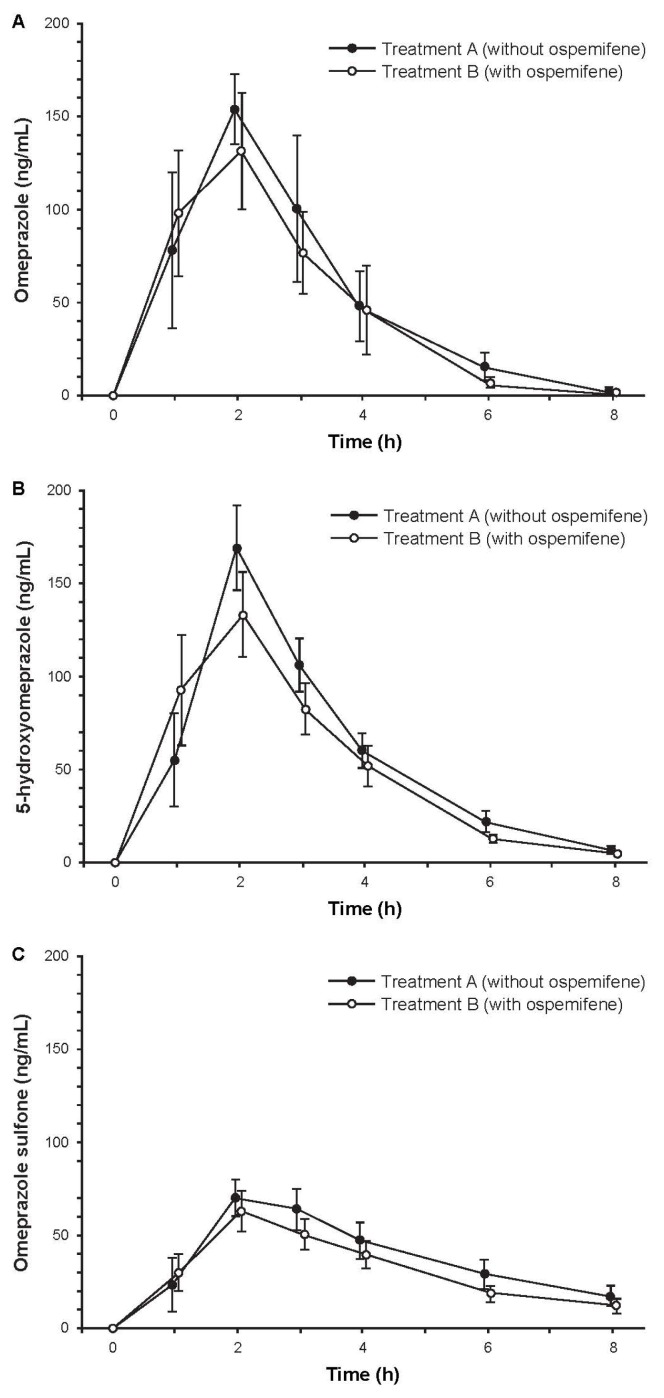
Mean concentration-time curves of plasma (**a**) omeprazole; (**b**) 5-hydroxyomeprazole, and (**c**) omeprazole sulfone after administration of 20 mg of omeprazole with and without ospemifene pretreatment. Error bars are ±SE, *N* = 12.

Subjects (*N* = 14) were Finnish white females with a mean age of 59.6 years (range, 50–67 years). Among the 12 subjects who completed the study, treatment compliance was 100%. Mean concentration-time curves for omeprazole, 5-hydroxyomeprazole, and omeprazole sulfone were similar with and without ospemifene pretreatment (Figure 3). The ratios of geometric least squares means with and without ospemifene treatment for omeprazole/5-hydroxyomeprazole and omeprazole/omeprazole sulfone based on 3-h plasma concentrations were close to one, indicating that ospemifene did not influence the metabolic ratios of omeprazole and its two metabolites (Table 3). However, the 90% CIs were not entirely within the pre-specified 80% to 125% acceptance range, due to high variability, and thus, bioequivalence could not be concluded. In contrast, the corresponding ratios of geometric least square means based on AUC*_t_*, which captures the sampling time from 0 to 8 h, were close to one and had 90% CIs that were within the pre-specified acceptance range (Table 3). These results indicated bioequivalence (absence of a pharmacokinetic interaction) with and without ospemifene pretreatment.

#### 2.3.4. Overall Safety

All treatments in the clinical studies (warfarin, bupropion, and omeprazole with and without ospemifene) were well tolerated. The most frequently reported treatment-emergent AE was headache. In the warfarin and bupropion studies, no subjects discontinued. In the omeprazole study, one subject discontinued before receiving study medication because of personal reasons; another subject received all doses of ospemifene in the first treatment period, but withdrew because of urinary tract infection, which was considered unrelated to study treatment.

The three clinical studies in healthy postmenopausal women demonstrated minimal drug-drug interactions of ospemifene with drug substrates (warfarin, bupropion, and omeprazole) metabolized by CYPs, with no clinically relevant changes in their pharmacokinetic parameters. Despite some *in vitro* interactions of ospemifene and its hydroxylated metabolites with CYP2B6-, CYP2C9-, CYP2C19-, and CYP3A4-mediated drug metabolism, the clinical study results indicated no effect of ospemifene on most pharmacokinetic parameters related to CYP2C19 and CYP3A4, and supported *in vitro* findings whereby ospemifene only weakly inhibited CYP2C19 activity and had no effect on CYP3A4 activity. Furthermore, in spite of the potency of 4-hydroxyospemifene to inhibit CYP2C9 in human liver microsomes *in vitro*, the *in vivo* data did not provide evidence of inhibition of CYP2C9 metabolism by ospemifene or its metabolites. Finally, there was no evidence that ospemifene would affect CYP2B6-mediated drug metabolism. These *in vivo* results are in agreement with the *in vitro* data demonstrating weak inhibition potential of ospemifene or its two major metabolites on CYP2B6 activity.

The results of these three clinical studies of ospemifene pretreatment on the pharmacokinetics of warfarin, bupropion, and omeprazole stand in partial contrast to the situation with other SERMs. Coadministration of tamoxifen with warfarin has led to bleeding complications, potentially due to inhibition by tamoxifen of CYP2C9-mediated metabolism of warfarin [12]. Toremifene is a weak inhibitor of CYP2C9, and it is recommended that patients receiving concomitant treatment with warfarin and other CYP2C9 substrates with a narrow therapeutic index should be carefully monitored [13]. Raloxifene is not metabolized by CYP enzymes [14] and thus is unlikely to have CYP-mediated effects on the pharmacokinetics of other drugs, although it did decrease the prothrombin time of patients on warfarin by 10% [15]. As with ospemifene, clinically significant effects on the pharmacokinetics of bupropion and omeprazole have not been observed with tamoxifen, toremifene, and raloxifene.

Although ospemifene did not have clinically significant effects on the pharmacokinetics of the three probe drugs tested in the clinical studies described in this article, other crossover studies (reported separately) that examined the possible effects of CYP inhibitors and inducers on the pharmacokinetics of ospemifene (data on file) demonstrated that serum concentrations of ospemifene were reduced with rifampicin pretreatment, increased with ketoconazole or fluconazole pretreatment, and minimally changed with omeprazole pretreatment. Thus, strong CYP3A or CYP2C9 inducers such as rifampicin would be expected to decrease the exposure to ospemifene; coadministration of the CYP3A inhibitor ketoconazole with ospemifene should be undertaken with caution; and the potent CYP3A/CYP2C9/CYP2C19 inhibitor fluconazole should not be coadministered with ospemifene. The potent CYP2C19 inhibitor omeprazole is unlikely to affect the pharmacokinetics of ospemifene to a clinically relevant degree.

## 3. Experimental Section

### 3.1. *In Vitro* Metabolism

#### 3.1.1. Reagents

Ospemifene and its two main metabolites, 4-hydroxyospemifene and 4′-hydroxyospemifene, were supplied by Hormos Medical Ltd. (Turku, Finland). All other reagents were obtained from commercial suppliers.

#### 3.1.2. Human Liver Microsomal Preparations

Human liver microsomes were prepared and pooled from ten human livers (HL20–24,28–32) as previously described [16]. Human liver samples were obtained from the University Hospital of Oulu as surplus tissue from cadaver kidney transplant donors. The collection of surplus tissue was approved by the Ethics Committee of the Medical Faculty of the University of Oulu, Finland. All donors were Caucasians ranging in age from 21 to 62 years; four were female and six were male. Detailed information on each donor has been published previously [16]. Protein concentrations were measured using the Bradford method [17]. Each microsome preparation was characterized with CYP-specific substrates and reference inhibitors; only those microsomal preparations whose activities were specifically inhibited were included into the pool, which was used for the preliminary screening of inhibition by the N-in-one assay.

#### 3.1.3. CYP Inhibition Assay

*In vitro* inhibition of CYP isoforms was first assessed using the N-in-one approach (also called the cocktail approach) [18,19]. CYP-selective substrates and reactions are listed in Table 1; concentrations were roughly at or below the respective Michaelis-Menten coefficients. Therefore, the IC_50_ values obtained for the tested inhibitors can be considered to approximate the results that would be derived from quantitative enzyme kinetic determinations. A Micromass LCT time-of-flight high-resolution mass spectrometer (Micromass Ltd., Manchester, UK) equipped with a Z-spray electrospray ionization source was used for the monitoring of CYP-selective reactions. Integrated mass chromatogram peak areas were normalized *versus* internal standards before comparing metabolites in different preparations. More details of the assay are available in previous publications [18,19].

#### 3.1.4. Determination of Inhibition Constants

IC_50_ values for ospemifene and 4-hydroxyospemifene were determined graphically by linear regression analysis of the logarithmic plot of inhibitor concentration *versus* percentage of activity remaining after inhibition using Microcal Origin, version 4.10 (Microcal Software, Inc., Northampton, MA, USA). Enzyme activities were compared with control incubations.

The type of inhibition of ospemifene toward CYP2B6-, CYP2C9-, CYP2C19-, CYP2D6- and CYP2C8-associated activities was determined by measuring four appropriate concentrations of ospemifene (selected on the basis of the screening phase) at a minimum of three different concentrations of the respective CYP-specific substrate (*K*_m_/2, *K*_m_, and 4 × *K*_m_). Dixon, Lineweaver-Burk, Hanes, and Hofstee plots were used for the analysis of the type of inhibition. Different plots were in agreement with each other.

#### 3.1.5. Assessment of CYP Induction in Isolated Human Hepatocytes

Freshly isolated human hepatocytes were obtained from the UK Human Tissue Bank (Leicester, UK). Hepatocyte viability was assessed using the trypan blue exclusion test (73%–86%). Hepatocytes were diluted in pre-incubation medium (William’s Medium E (without phenol red) supplemented with 0.1 μM dexamethasone, 0.0001% insulin, 100 IU/mL penicillin, 100 μg/mL streptomycin, and 10% *v*/*v* fetal bovine serum or newborn calf serum) and incubated in a humidified incubator for 2 to 4 h at 37 °C in an atmosphere of 95% air/5% CO_2_. Pre-incubation medium was replaced with incubation medium (pre-incubation medium minus serum) and cells were incubated for an additional 24 to 36 h at 37 °C.

Next, fresh incubation media containing ospemifene at concentrations of 0.2, 2, or 20 μM was added to cell cultures and incubated for a further 48 h with medium changes at 24-h intervals. Omeprazole (CYP1A2 inducer; 30 μM), phenobarbital (CYP2B6 inducer; 1 mM) and rifampicin (CYP2C9, CYP2C19, and CYP3A4 inducer; 50 μM) dissolved in dimethyl sulfoxide (0.5% *v*/*v* final), served as reference inducers. At the end of this 48-h incubation period, cells were incubated for 1 h in media containing CYP substrates in various combinations: activities of 7-ethoxyresorufin *O*-deethylase (CYP1A2 probe; 5 μM), bupropion hydroxylase (CYP2B6 probe; 500 μM), diclofenac 4′-hydroxylase (CYP2C9 probe; 100 μM), *S*-mephenytoin hydroxylase (CYP2C19 probe; 150 μM), and [^14^C]testosterone 6β-hydroxylase (CYP3A4 probe; 250 μM) were determined using fluorometric, liquid chromatography/mass spectrometry (LC/MS), or radiochromatographic methods according to established protocols.

### 3.2. *In Vivo* Drug Interaction Studies

All experiments with human subjects were based on voluntary informed consent and the clinical study protocols had appropriate ethical and regulatory approval. Subjects were asked to immediately report any significant adverse events (AEs) that occurred during the study; minor AEs were reported at clinic visits. AEs were coded and classified according to the Medical Dictionary for Regulatory Activities.

Studies 1 and 2 were open-label, two-period, single-center, Phase 1, balanced, crossover studies in healthy postmenopausal women. Subjects had not taken medications known to affect CYP2C9 or CYP3A4 metabolism within four weeks of the start date (study 1) or were not homozygous for the CYP2B*6 genotype (CYP2B*6 indicates the CYP2B6 variant that is associated with reduced activity; study 2). Study 3 was a Phase 1, open-label, single-center, balanced, two-period, crossover study in healthy postmenopausal women who were not homozygous for the CYP2C19*2 genotype (CYP2C19*2 indicates the CYP2C19 variant that is associated with reduced activity). In each study, equal numbers of subjects were assigned to the two different treatment sequences.

Study 1: In one of the study periods, the subjects were pretreated with once-daily oral ospemifene (60 mg) for 12 days, and were given a single dose of 10 mg of racemic warfarin and 10 mg of vitamin K after an overnight fast on the morning of study day 8. In the other study period, 10 mg of racemic warfarin and 10 mg of vitamin K were given without ospemifene pretreatment. The study periods were separated by ≥3 weeks. In both study periods, blood samples for determination of plasma warfarin concentrations were collected before and 2, 4, 6, 8, 12, 24, 48, 72, 96, and 120 h after warfarin administration.

Study 2: In one of the study periods, the subjects were pretreated with once-daily oral ospemifene (60 mg) for 8 days, and were given a single 150 mg dose of bupropion after an overnight fast on day 8. In the other study period, 150 mg of bupropion was given without ospemifene pretreatment. The study periods were separated by ≥14 days. In both study periods, blood samples for determination of plasma bupropion and hydroxybupropion concentrations were collected before and 1, 2, 3, 4, 5, 6, 8, 12, 24, 32, 48, 56, 72, and 96 h after bupropion administration.

Study 3: In one of the study periods, the subjects were pretreated with once-daily oral ospemifene (60 mg) for 7 days, and were given a single dose of 20 mg of omeprazole after an overnight fast on day 8. In the other study period, 20 mg of omeprazole was given without ospemifene pretreatment. The study periods were separated by ≥14 days. In both study periods, blood samples for determination of plasma omeprazole, 5-hydroxyomeprazole, and omeprazole sulfone concentrations were collected before and at 1, 2, 3, 4, 6, and 8 h after omeprazole administration.

#### Pharmacokinetic Parameters

In Studies 1 and 2, concentrations of *S*-warfarin, bupropion, and hydroxybupropion were determined using validated LC-MS/MS methods. Pharmacokinetic parameters of *S*-warfarin, bupropion, and hydroxybupropion were calculated from the concentration-time data of both treatment periods using the WinNonlin Professional software package (Pharsight Corporation, Mountain View, CA, USA) and noncompartmental methods. With the exception of *t*_max_, which was analyzed using the nonparametric Wilcoxon’s test, pharmacokinetic parameters were analyzed after log-transformation using analysis of variance (ANOVA), with sequence, period, and treatment as fixed effects. The primary pharmacokinetic parameter was the mean area under the plasma concentration-time curve from time 0 to infinity (AUC_∞_) of *S*-warfarin (study 1) or hydroxybupropion (study 2). Bioinequivalence was indicated by 90% CIs for the ratios of AUC_∞_ that were outside the range of 0.80 to 1.25.

In Study 3, concentrations of omeprazole, 5-hydroxyomeprazole, and omeprazole sulfone were determined using a validated LC-MS/MS method. Pharmacokinetic parameters were analyzed after log-transformation using ANOVA, with sequence, period, and treatment as fixed effects. Primary pharmacokinetic parameters included the metabolic ratios of omeprazole/5-hydroxyomeprazole and omeprazole/omeprazole sulfone, based on plasma concentrations at 3 h, which reflected CYP2C19 and CYP3A4 activities, respectively. The metabolic ratios based on AUC calculated from 0 to 8 h (AUC*_t_*) were the key secondary parameters. Bioinequivalence was indicated by 90% CIs for the ratios of primary and secondary parameters that were outside the range of 0.80 to 1.25.

## 4. Conclusions

Results from these studies indicate low potential for ospemifene to alter the metabolism or pharmacokinetics of drugs that are CYP substrates. There were no clinically relevant changes in pharmacokinetic parameters for warfarin, bupropion, or omeprazole. The results obtained in the preclinical *in vitro* assays were judged to demonstrate a relatively weak inhibitory potency of ospemifene and indeed, this expectation was confirmed by targeted clinical studies.

## Figures and Tables

**Table 1:** Comparison of inhibitory potencies of ospemifene, 4-hydroxyospemifene, and 4′-hydroxyospemifene as measured using the current N-in-one assay in human liver microsomes *in vitro*.

Enzyme	Model reaction (probe concentration)	IC_50_ (μM)		

		Ospemifene	4-Hydroxy-ospemifene	4′-Hydroxy-ospemifene
CYP1A2	Melatonin 6-hydroxylation (4 μM)	>100	>100	>100
CYP2A6	Coumarin 7-hydroxylation (2 μM)	>100	>100	>100
CYP2B6	Bupropion hydroxylation (1 μM)	7.8	26.5	15
CYP2C8	Amodiaquine desethylation (2 μM)	36.4	27.7	7
CYP2C9	Tolbutamide methylhydroxylation (4 μM)	10	1.1	12
CYP2C19	Omeprazole 5-hydroxylation (2 μM)	22.5	15.3	50
CYP2C19	Omeprazole demethylation (2 μM)	47.2	19.7	50
CYP2D6	Dextromethorphan O-demethylation (0.2 μM)	48.7	25.5	86
CYP2E1	Chlorzoxazone 6-hydroxylation (6 μM)	>100	>100	>100
CYP3A4	Midazolam 1′-hydroxylation (0.4 μM)	>100	>100	78
CYP3A4	Testosterone 6β-hydroxylation (1 μM)	>100	>100	>100
CYP3A4	Omeprazole sulfoxidation (2 μM)	>100	35.9	85
CYP3A4	Omeprazole 3-hydroxylation (2 μM)	37.9	50.0	54

CYP = cytochrome P450; IC_50_ = half maximal inhibitory concentration.

**Table 2:** Mean (CV%) pharmacokinetic parameters of *S*-warfarin, *R*-warfarin, bupropion, and hydroxybupropion with and without ospemifene pretreatment.

Parameter	*S*-Warfarin (*N* = 16)		*R*-Warfarin (*N* = 16)		Bupropion (*N* = 16)		Hydroxybupropion (*N* = 16)	
			
	OSP (−)	OSP (+)	OSP (−)	OSP (+)	OSP (−)	OSP (+)	OSP (−)	OSP (+)
AUC_∞_ (μg h/mL)	18.5 (29.9)	19.2 (30.2)	31.3 (21.7)	32.8 (23.1)	0.90 (32.3)	0.75 (38.5)	15.5 (29.4)	15.2 (32.4)

AUC_t_ (μg h/mL)	17.2 (27.4)	18.0 (28.3)	26.2 (18.7)	27.8 (19.6)	0.85 (33.7)	0.70 (38.5)	14.3 (28.1)	14.5 (31.7)

*C*_max_ (ng/mL)	497 (14.5)	513 (13.3)	518 (15.2)	538 (15.4)	74.9 (36.6)	62.9 (40.4)	398 (30.6)	462 (29.0)

*t*_max_ (h) †	4 (2–6)	4 (2–8)	4 (2–6)	4 (2–8)	3.5 (1–5)	3.0 (1–5)	6.0 (5–24)	6.1 (5–8)

*t*_1/2_ (h)	30.4 (13.2)	29.2 (12.7)	43.8 (17.1)	42.7 (15.6)	30.8 (29.4)	29.5 (26.7)	26.7 (18.8)	21.2 (18.0)

CL/F (mL/h)	587 (29.2)	564 (27.5)	334 (21.2)	320 (23.0)	185 (38.1)	230 (38.1)	n.d.	n.d.

† Median;

AUC_∞_ = area under the plasma concentration-time curve extrapolated to infinity; AUC_t_ = area under the plasma concentration-time curve from zero to the last sampling time point (120 or 96 h); CL/F = oral clearance; *C*_max_ = maximal concentration; CV% = coefficient of variation; n.d. = not determined; OSP = ospemifene; *t*_max_ = time to reach *C*_max_; *t*_1/2_ = half-life.

**Table 3:** Geometric mean (CV%) and equivalence statistics of the metabolic ratios of omeprazole and its metabolites (omeprazole sulfone and 5-hydroxyomeprazole) with and without ospemifene pretreatment.

	Metabolic ratio	Ospemifene (−) (*N* = 12)	Ospemifene (+) (*N* = 12)	Ratio * (90% CI) (*N* = 12)
3 h conc.	Omeprazole/5-hydroxyomeprazole	0.67 (78.4)	0.65 (98.7)	0.97 (0.77–1.22)
	Omeprazole/Omeprazole sulfone	1.19 (52.7)	1.15 (100.9)	0.97 (0.67–1.41)
AUC*_t_*	Omeprazole/5-hydroxyomeprazole	0.78 (59.0)	0.76 (61.5)	0.97 (0.88–1.08)
	Omeprazole/Omeprazole sulfone	1.36 (20.4)	1.37 (24.5)	1.00 (0.88–1.15)

* Ratio of ospemifene (+)/ospemifene (−).

AUC*_t_* = area under the plasma concentration-time curve from 0 to 8 h; CI = confidence interval; conc = concentration.
